# Semisupervised representative learning for measuring epidermal thickness in human subjects in optical coherence tomography by leveraging datasets from rodent models

**DOI:** 10.1117/1.JBO.27.8.085002

**Published:** 2022-08-19

**Authors:** Yubo Ji, Shufan Yang, Kanheng Zhou, Jie Lu, Ruikang Wang, Holly R. Rocliffe, Antonella Pellicoro, Jenna L. Cash, Chunhui Li, Zhihong Huang

**Affiliations:** a University of Dundee, School of Science and Engineering, Dundee, United Kingdom; b Edinburgh Napier University, School of Computing, Edinburgh, United Kingdom; c University of Glasgow, Center of Medical and Industrial Ultrasonics, Glasgow, United Kingdom; d University of Washington, Department of Bioengineering, Seattle, Washington, United States; e The University of Edinburgh, The Queen’s Medical Research Institute, MRC Centre for Inflammation Research, Edinburgh, United Kingdom

**Keywords:** optical coherence tomography, semisupervised learning, acute burning wound, re-epithelialization, epidermis, scab

## Abstract

**Significance:**

Morphological changes in the epidermis layer are critical for the diagnosis and assessment of various skin diseases. Due to its noninvasiveness, optical coherence tomography (OCT) is a good candidate for observing microstructural changes in skin. Convolutional neural network (CNN) has been successfully used for automated segmentation of the skin layers of OCT images to provide an objective evaluation of skin disorders. Such method is reliable, provided that a large amount of labeled data is available, which is very time-consuming and tedious. The scarcity of patient data also puts another layer of difficulty to make the model more generalizable.

**Aim:**

We developed a semisupervised representation learning method to provide data augmentations.

**Approach:**

We used rodent models to train neural networks for accurate segmentation of clinical data.

**Result:**

The learning quality is maintained with only one OCT labeled image per volume that is acquired from patients. Data augmentation introduces a semantically meaningful variance, allowing for better generalization. Our experiments demonstrate the proposed method can achieve accurate segmentation and thickness measurement of the epidermis.

**Conclusion:**

This is the first report of semisupervised representative learning applied to OCT images from clinical data by making full use of the data acquired from rodent models. The proposed method promises to aid in the clinical assessment and treatment planning of skin diseases.

## 1 Introduction

The skin is the largest organ of the human body and serves essential functions to maintain homeostasis of the body. The epidermal homeostasis can be disrupted by various skin diseases, which often cause morphological changes in the epidermis.^1^^,^^2^ Epidermal thickness is critically important information in the assessment and treatment planning of a variety of dermatologic conditions. Traditional invasive modality, e.g., biopsy, can cause complications such as bleeding, infection, and scarring.^3^^–^^5^

Being a noninvasive three-dimensional (3D) optical imaging modality, optical coherence tomography (OCT) has recently attracted attention with respect to its use in assessing a range of skin conditions, including wound healing,^6^ acne lesion,^7^^,^^8^ papule,^9^ and skin cancer.^10^ It has the potential to serve as a nontraumatic alternative to the biopsy. However, due to the complicated morphological change within the variety of skin diseases, accurate annotation of epidermis structure based on OCT is a challenging task even for experienced experts.^11^ Moreover, manual segmentations are extremely time-consuming with variable interpretation, repeatability, and interobserver agreement, which is not suitable for clinical applications. Traditional OCT epidermal segmentation is based on Shapelet-based image processing^12^ or detection of minimum local intensity of the dermal-epidermal junction.^13^ Lu et al.^14^ developed a simple and efficient method by estimating the attenuation coefficient from OCT signals of burned wound to enhance the contrast between the dermis and epidermis. However, the segmentation process proposed by those researchers highly relies on the image quality, and it is prone to segmentation errors if large variances of skin pathologies based on OCT are present. The deep convolutional neural networks (CNNs) have achieved great success in the segmentation tasks due to their ability to learn high-level task-specific imaging features.^15^^,^^16^ Nevertheless, these methods are supervised learning and rely on large-scale labeled data. The accuracy and generalizability of the model are greatly limited by the lack of datasets in patients.^17^ Due to the difficulty of obtaining sufficient labeled data, researchers are looking for self-supervised learning.

Considering the similar optical scattering features between the human and mouse layer, we can leverage knowledge from pretrained mouse models and use it for prediction of patient data. Nevertheless, this task is quite challenging for several reasons. First, there are significant interspecies differences in the anatomy and physiology of each layer, which greatly differ in thickness. Mouse epidermis generally comprises only three cell layers and is <25  μm in thickness, whereas human epidermis commonly constitutes 6 to 10 cell layers and is >50-μm thick.^18^ Human skin is firm, and adhered to the underlying tissues, whereas murine skin is loose.^19^ Second, it has been demonstrated that variations in structural features occurred within the different anatomic sites, age,^20^^,^^21^ and lesion types, e.g., epidermal thickening within the active plaque in psoriasis while disarranged epidermal pattern in squamous cell carcinoma (SCC).^22^^,^^23^ In such cases, methods utilizing the prior knowledge of mouse model are prone to fail in prediction of human data. Therefore, finding a proper solution to bridge the gap between mouse OCT data and human OCT data is in high demand to improve the efficiency as well as reliability in the clinical practice. Self-supervised representation learning is based on the understanding of how to encode the trained network in the source domain to recognize slightly altered images in the target domain. The source domain in this study is built on the mouse skin data and the target domain is based on patients’ data. As human and mouse skin have similar cellular compositions in the dermis and epidermis, rodent model has been employed to mimic human skin disorder based on OCT in recent years to derisk clinical trials on humans.^24^^–^^28^ The OCT data based on rodent models are typically abundant due to there being more accessibility, allowing the use of a relatively high number of animals for statistical validation.^19^^,^^27^^–^^31^

One explosive space of research in the self-supervised representation learning is contrastive learning, which has gained increasing attention by showing robust performance for image segmentation using the learned representation in ImageNet.^32^^–^^34^ However, the quality of learned representation depends on the effort of developing effective data augmentation by contrasting latent representation of different augmentations.^35^ The most common research is only focused on comparing representation learned by self-supervised methods on pretrained models from ImageNet, such as MoCo^36^ and SimCLR.^37^ Contrastive learning attempts to keep the balance of the variance and entanglement in the latent space, which indicates the similarity of image features and relationship between true representations and noise information. Generally speaking, the contrastive noise is used to guide learned representation to map true pairs and nearby locations; negative pair is to pull them apart. Since there is no supervision in contrastive learning, it is difficult to associate the successful invariant to all but one type of data augmentation.

In this work, we propose an easy and efficient strategy for utilizing the mouse annotation data to serve on clinical OCT datasets acquired from burn patients. A novel semisupervised representation learning method is described, in which the automatic segmented epidermis at various morphological states with only one labeled image per OCT volume is needed from human subjects. Then, the *en-face* epidermal thickness mapping and its quantitative analysis are provided for elucidation of the epidermal response to burn injury over time in patients. We verify our method on clinical data for three stages of acute burned skin as well as normal control data and compare with three data augmented strategies to evaluate the performance of segmentation using U-Net encoding. We believe a latent space transformation incurs a spectrum of robustness to build a connection among interspecies samples from OCT scans. The approach can achieve clinically valid performance with only one labeled image per volume from patients’ datasets. To our knowledge, this is the first application of semisupervised representation learning for automatic OCT image segmentation in clinical datasets by making full use of the datasets acquired from rodent models.

## 2 Semisupervised Representation Learning

**Fig. 1:**
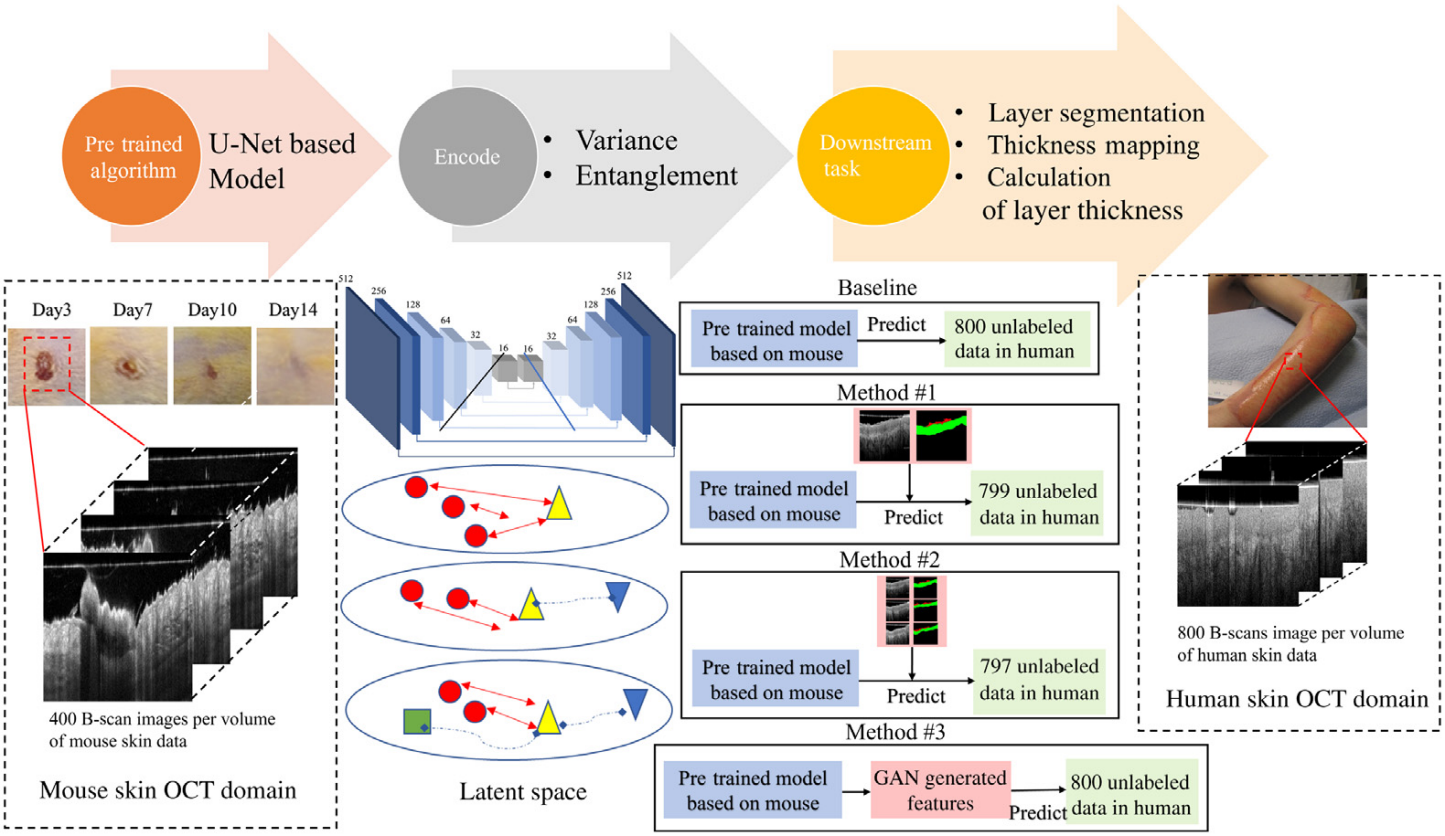
Illustration of proposed semisupervised learning. First, the U-Net pretrained model is utilized in source domain obtained by the rodent model. Second, trained network is encoded using three different augmented strategies (methods #1, #2, and #3). Green box indicates the information in source domain, yellow triangle indicates the information in target domain, red circles represent the noise information, blue upside-down triangle is the similarity between source and target. Last, an updated parameter is applied to predict human clinical datasets where epidermis thickness mapping and averaged epidermal thickness are obtained automatically.

Our proposed semisupervised architecture can be divided into three steps (see Fig. 1): first, the multitask U-Net is used to pretrain the model in the source domain (rodent data); second, the pretrained model was encoded to keep the feature map invariance and entanglement using semisupervised representation; finally, at the end-task stage, we adapt the parameters on pixel-wise features and sematic segmentation to the target domain (human data), where epidermis thickness mapping and averaged epidermal thickness are obtained automatically.

The latent space in Fig. 1 represents the illustration of a semisupervised training method. Semi-supervised learning is halfway between the supervised and unsupervised learning. In addition to the unlabeled data, the algorithms are provided with some supervised information. In the latent space at the encoding stage, the yellow triangle indicates those target domains. The red circles indicate the noise information. The similarity forms a blue upside-down triangle that pulls the target domain. Method #1 only randomly selects one labeled image from each volume (800 B-scan images). Method #2 used three labeled images from each volume. The last one (method #3) used cycle-GAN architecture formed synthetic data to increase the invariance encoding spaces using cycle-consistency loss function in combination with adversarial loss.^38^ For comparison, the result of directly applying the pretrained rodent model to predict human data was performed as baseline.

**Fig. 2:**
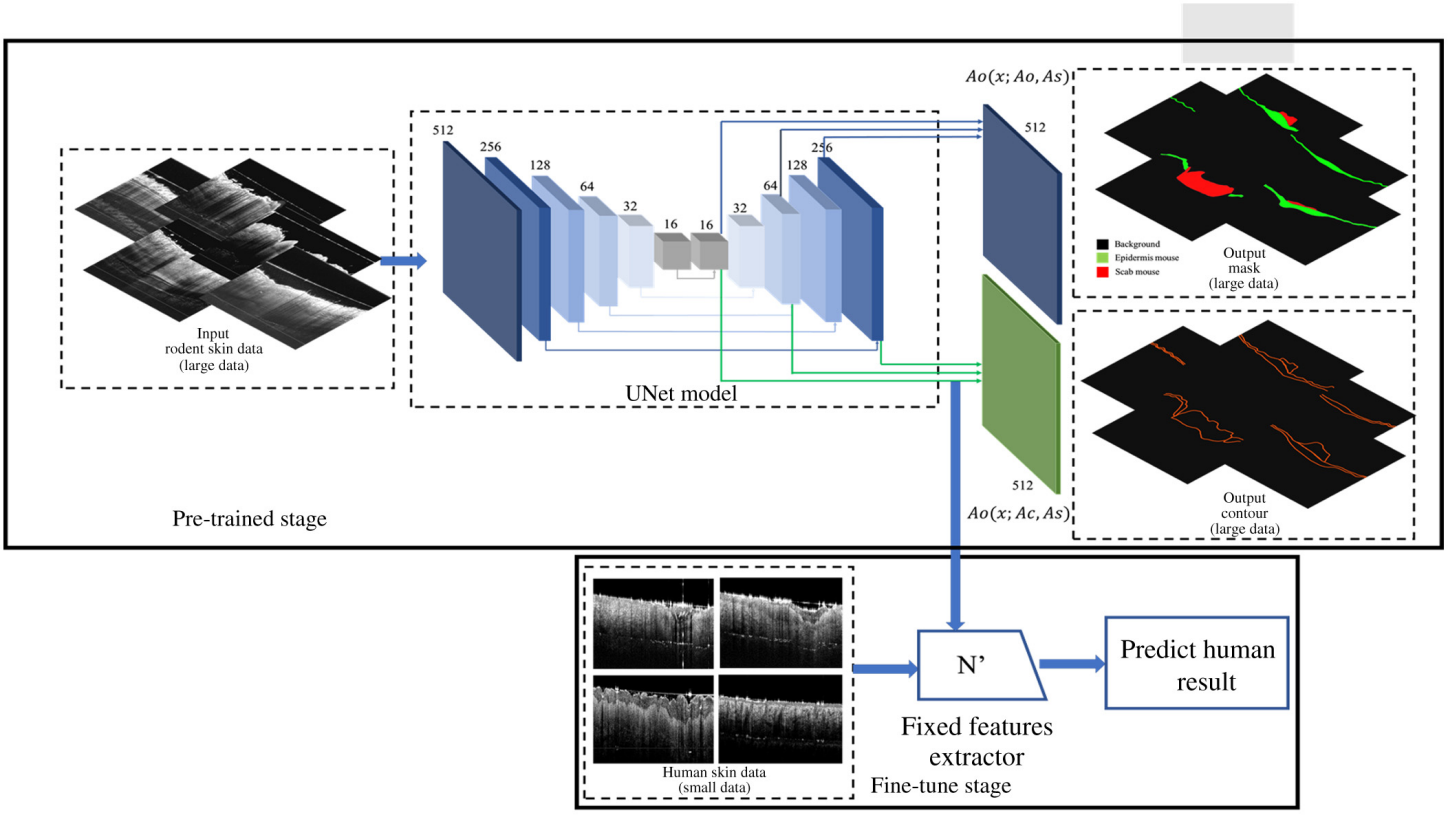
Architecture of U-Net multitask model.

The scab is an interference structure when segmenting the epidermis as it is always located at the top of the epidermis. In this study, the structure of epidermis and scab were segmented simultaneously. We formulated the model as a semisupervised learning framework and multitask model by inferring the information of contours and objects simultaneously. The pretrained multitask model is illustrated in Fig. 2. The backbone U-Net^39^ is selected as the base architecture in the model to build encoding. Each encoder block is made of four layers, which are arranged in the following order: convolution layer, batch normalization (BN) layer, ReLU activation layer, and max-pooling layer. Each down-sampling included two 3×3 convolutions, BN and Leaky ReLU, and a 2×2 max-pooling with a stride of 2. Each decoder block is made up of five layers, which are listed in the following order: unpooling layer, concatenation layer, convolution layer, BN layer, and ReLU activation function. Each step of up-sampling contained one 2×2 deconvolution with a stride of 2 and two 3×3 convolutions followed by BN and Leaky ReLU. For end-task, to output the segmentation masks of intensity and contours information, the parameters of down sampling path As are shared and updated for these two tasks jointly. An entropy minimization method is used to abstract the featured representations through the hierarchical structure.

In step 1, the training of the network is expressed as a per-pixel classification issue, which is similar to^40^

$$L_{\text{total}}(x;\theta )=\lambda \psi (\theta )-\underset{x\in X}{\sum }\;\log\;N_{o}(x,l_{o}(x);A_{O},A_{s})-\underset{x\in X}{\sum }\;\log\;N_{c}(x,l_{c}(x);A_{O},A_{s}),$$ (1)

where the first item is the $L_{2}$ regularization and the latter two items represent error loss. x is the pixel position in image space X, $N_{o}(x,l_{o}(x);A_{O},A_{S})$ denotes the predicted probability for true label $l_{o}(x)$ of epidermis and scab objects after softmax classification, and $N_{C}(x,l_{C}(x);A_{C},A_{C})$ is the predicted probability for true label $l_{C}(x)$ of the skin layer contours. The parameters $\theta =\{A_{s},A_{O},A_{c}\}$ of the network are optimized by minimizing the total loss function $L_{\text{total}}$ with standard backpropagation.

The complementary information is fused together to construct the final segmentation masks s(x) using the predicted probability maps of structural object $N_{O}(x;A_{O},A_{S})$ and contour $N_{c}(x;A_{C,}As)$ from the deep contour-prior network, defined as

$$s(x)=\{\begin{array}{ll} 2(\text{Scab}) & \text{if }N_{o}(x;A_{o},A_{s})<t_{o1}\text{ and }N_{c}(x;A_{c},A_{s})<t_{c1}\\ 1(\text{Epidermis}) & \text{if }t_{o1}\leq N_{o}(x;A_{o},A_{s})<t_{o2}\text{ and }N_{c}(x;A_{c},A_{s})<t_{c2}\\ 0 & \text{Otherwise} \end{array},$$ (2)

where $t_{O1}$, $t_{O2}$, $t_{c1}$, and $t_{c2}$ are the threshold (set as 0.5) in our experiments empirically.

Step 2 of our DL-based architecture involves transfer learning (TL), which updates the parameter of the pretrained multitask model using the ADAM optimizer and loss function as

$$L_{\text{Transfer}}=-\overset{M}{\underset{c=1}{\sum }}y_{c}\;\log(p_{c}),$$ (3)

where M indicates the number of classes (three in this study) and $y_{c}$ represents the three-class one-hot coding vectors. $P_{C}$ represents the probability of an event estimated from the training set.

The last step is applying the updated parameters in U-Net architecture to clinical datasets. The epidermis thickness map was obtained by calculating the depth separation between the upper and lower boundaries of predicted structure by semisupervised networks at each A-line.

Step 1 was trained with 500 epochs using images in a pretrained rodent model. To fine-tune the model (step 2), we tested a variety of labeled human skin datasets, lowered the learning rate to 0.0001, and trained it for 50 epochs. The result is periodically assessed against the validation set (cross-validation). The time for training the pretrained model was 1.2 days and for self-supervised learning is ∼10  min using parallelized training across 12 × NVIDIA 1080ti GPUs with NVIDIA CUDA (v10.1).

## 3 Experiment

### 3.1 Data Collection

The system used for this study was an in-house-built, experimental prototype swept source-OCT (SS-OCT) system. This SS-OCT system was powered by a 200 KHz vertical-cavity surface-emitting (VCSEL) swept laser source (SL1310V1-20048, Thorlabs Inc., Newton, United States), which provides a central wavelength of 1310 nm (infrared/IR) and a spectral bandwidth of 100 nm, giving an axial resolution of ∼8  μm in tissue (∼11  μm in the air). The sample arm consisted of a hand-held probe, where a pair 2D galvo-scanner, objective lens, collimator, and display system (a mini charge-coupled device camera and a mounted screen) were housed.

All experiments for the source domain (murine model) were conducted with approval from the local ethical review committee at the University of Edinburgh and in accordance with the UK Home Office regulations (Guidance on the Operation of Animals, Scientific Procedures Act, 1986). Experiments on animals were performed under PIL I61689163 and PPL PD3147DAB (January 2018 to January 2021). Experiments were performed on 8-week-old male (8W) C57Bl/6J wild-type mice (Charles River Laboratories, Tranent, United Kingdom). Four full-thickness excisional wounds were made to the dorsal skin using a sterile, single-use 4-mm punch biopsy tool for each mouse (Kai Medical; Selles Medical, Hull, United Kingdom). Each mouse underwent OCT examinations on the days 3, 7, 10, and 14 postinjuries. Control data was acquired at the region adjacent to the wound area. Each scanning session for each mouse should be guaranteed to be blasted <25  min. The dataset in the source domain consisted of 80 OCT volumes from six healthy mice. The size of each data volume contains 400×400×1920  pixels, covering 0.4  cm×0.4  cm. Due to strong motion and shadow artifacts, seven volumes had to be excluded, resulting in a total number of 73 OCT volumes, consisting of 29,200 OCT cross-section images in total available for use in this study (73×400 number of B-scans).

**Fig. 3:**
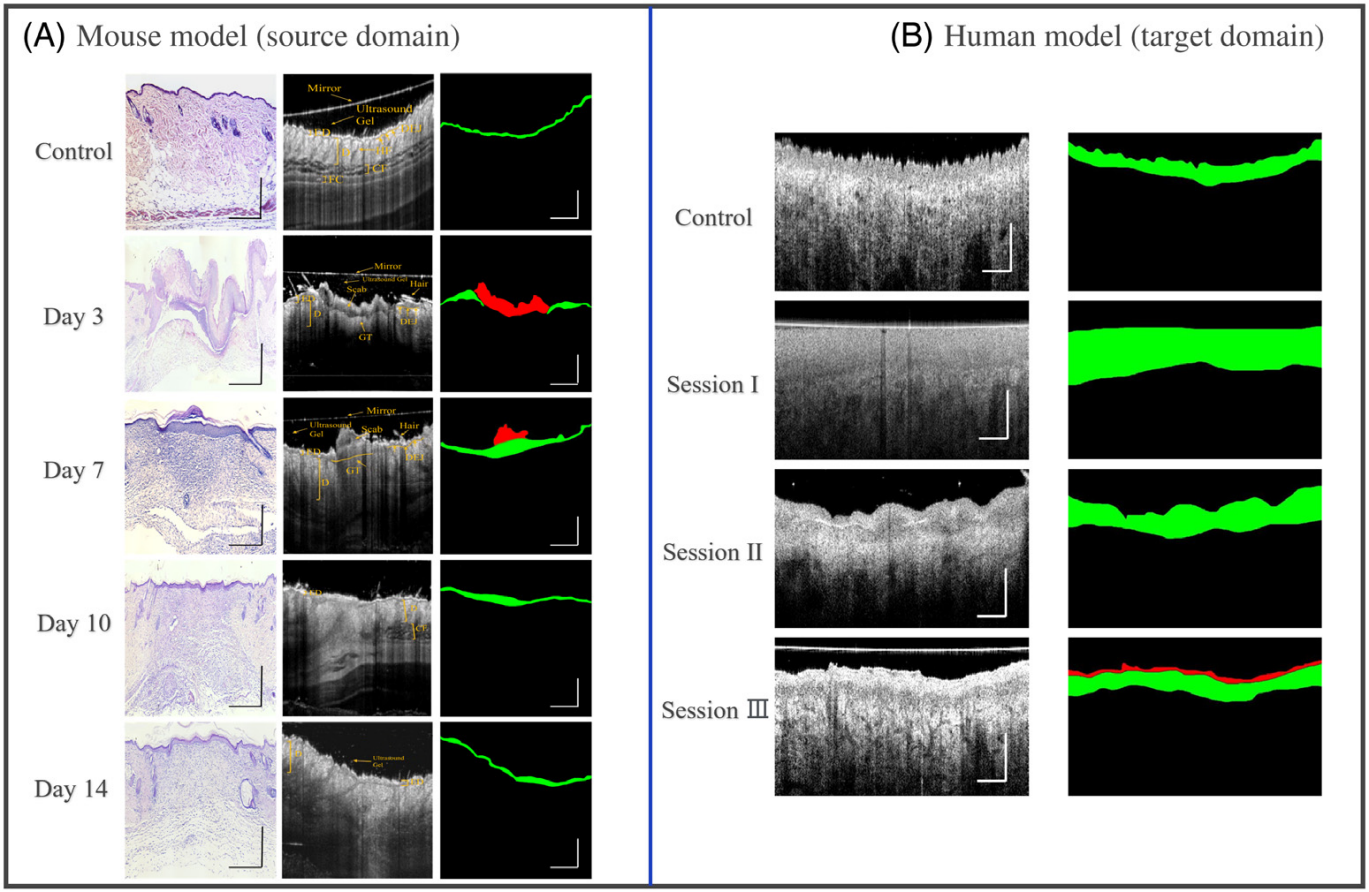
Scheme for data collection and images labeling manually in mouse and human. (a) Scheme of mouse model (source domain) segmentation; first column represents representative histology images (H&E staining) for control, wound healing day 3, 7, 10, and 14, respectively; second column is representative cross-section OCT images and structure annotation; third column is mask according to the manual segmentation contour where the red mask represents scab region, the green mask represents epithelial region, and black color represents remaining area including other anatomy structure and background information. (b) Scheme of human model (target domain) segmentation; first column is representative cross-section images at burning wound healing in sessions I, II, III, and control; second column is epidermis and scab mask similar with mouse. Scale bar represents 1 mm.

With the confirmation of its corresponding histology data and angiographic information, 2920 representative OCT B-scan images (200-control, 600-Day3, 640-Day7, 720-Day10, and 760-Day14) are manually labeled by two experts using a custom software by MATLAB [Fig. 3(a)]. Among them, 1460 images were used for training while the rest of 1460 images were utilized for cross-validation. Pixels in each B-scan image were divided into the following three classes: the red mask represents scab region while the green mask represents epidermis region, black color represents the remaining area including other anatomy structure and background information. Meanwhile, the contour of the object structure with 5-pixel width was generated automatically. Thus, the contour information was converted into a binary image where the pixel in the contour is 1 while the pixel out of the contour is 0.

The target domain was built based on the patients with acute burned wounds. All the experiments were approved by the Institutional Review Board (IRB) of University of Washington. The participants included in this study meet the following criteria: (i) age≥18  years; (ii) the size of the burning wound ≥1% total body surface area. In total, five patients (#2, #3, #4, #8, and #13) were included, and the written informed consent was obtained from all participants. To explore the physiological basis of human wound healing, all the participants were scanned at three image sessions at the time of wound healing stages: session I within 3 to 6 days, session II within 8 to 14 days, and session III within 19 to 22 days. The size of each data volume contains 800×800×1920  pixels, covering a region of 0.9  cm×0.9  cm. The acquisition time for a 3D scan was ∼6  s and the imaging session for each subject lasted ∼30  min. In total, 19 3D data volumes were obtained. The epidermis and scab region in human data was labeled by two experts using a method similar to mouse data annotation [see Fig. 3(b)]. Different sizes of labeled clinical datasets, 19 (one representative image for each volume), 57 (three representative images for each volume), and 0 (contrastive learning based on Cycle-Gan), were selected for self-supervised learning. Another 760 images were used for cross-validation while all the 15,200 images were used for testing to generate layer thickness mapping. The detailed sample size for training, TL, validation, and test in source domain and target domain is summarized in Table 1.

**Table 1:** Summary of overall data size of mouse skin OCT B-scans and human skin OCT B-scan for training, validation, and test.

	Source domain (mouse)	Target domain (human)
Data volumes (in total)	73 (5-control, 15-day3, 16-day7, 18-day10, and 19-day14)	19 (4-control, 5-session I, 5-session II, and 5-session III)
Labeled images by experts	2920	760
Data size for training	1460	
Data size for TL		19 (one image per volume)/57 (three images per volume)/0 (zero image per volume)
Data size for validation	1460	760
Data size for test	29,200 (73 × 400)	15,200 (19 × 800)

### 3.2 Implementation Details

**Fig. 4:**
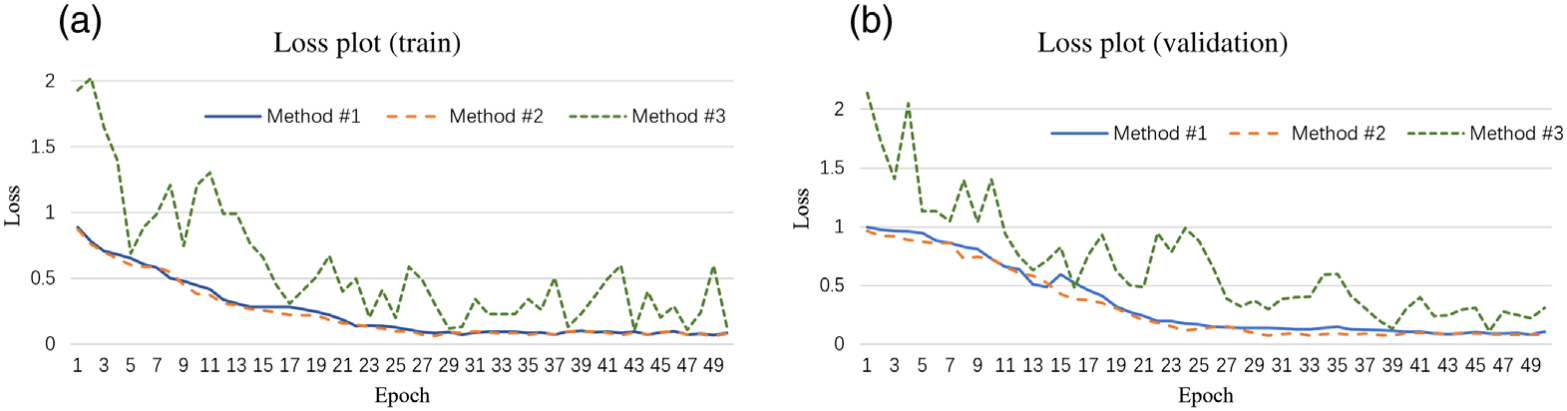
Training loss and validation loss plot of methods #1, #2, and #3.

Figure 4 summarized the training loss and validation loss in the semi-supervised learning of methods #1, #2, and #3. It demonstrated that both training loss and evaluation loss decreased at around 30 epochs and both smoothly down to the same level (∼0.15) for methods #1 and #2. It shows the model improves as its training and not obviously overfitting the training data. Method #3 provides a more fluctuating learning curve over epoch, which is important since utilizing Cycle-Gan to generate features without labeled data results in an unstable performance. After 40 epochs, the loss is fluctuated around 0.3 for method #3, which is slightly higher than methods #1 and #2.

## 4 Result

### 4.1 Qualitative Segmentation Accuracy Analysis

**Fig. 5:**
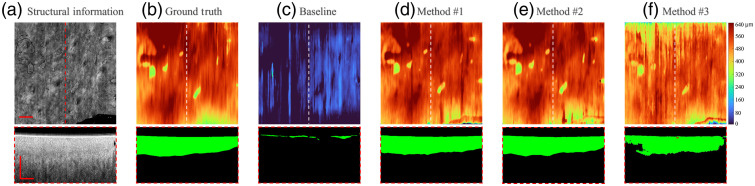
Segmentation results of OCT B-scan are in session I of patient #4. The representative B-scan OCT images and its corresponding *en-face* images are shown in (a), with expert annotations in (b), and baseline results in (c). The segmentation results for methods #1, #2, and #3 are shown in (d)–(f), respectively. The location of the selected cross-sectional images was indicated by the dashed lines. The scale bar=1  mm.

**Fig. 6:**
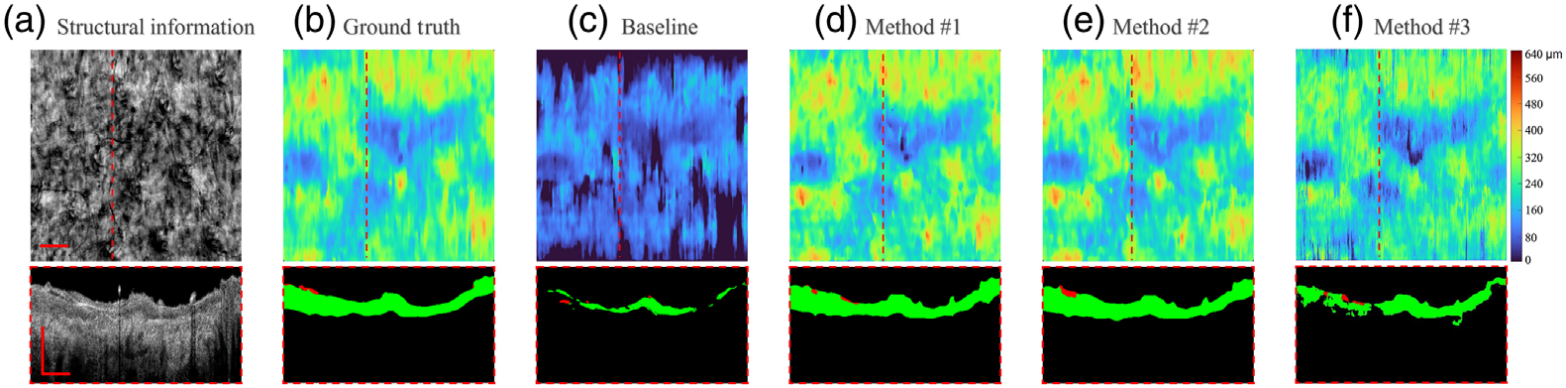
Segmentation results of OCT B-scan are in session II of patient #4. The representative B-scan OCT images and its corresponding *en-face* images are shown in (a), with expert annotations in (b) and baseline results in (c). The segmentation result for methods #1, #2, and #3 are shown in (d)–(f), respectively. The location of the selected cross-sectional images was indicated by the dashed lines. The scale bar=1  mm.

**Fig. 7:**
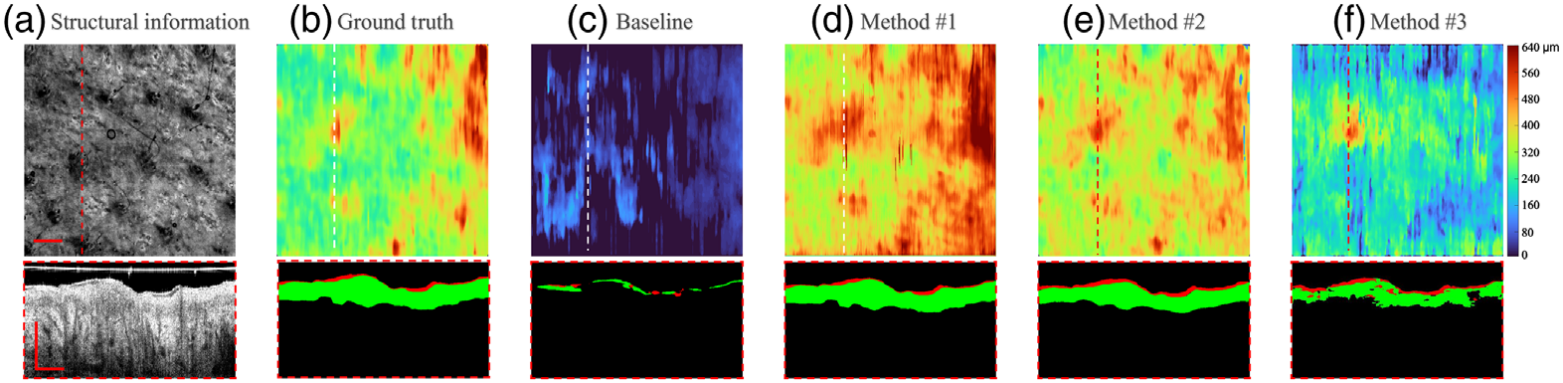
Segmentation results of OCT B-scan are in session III of patient #4. The representative B-scan OCT images and its corresponding *en-face* images are shown in (a), with expert annotations in (b) and baseline results in (c). The segmentation results for methods #1, #2, and #3 are shown in (d)–(f), respectively. The location of the selected cross-sectional images was indicated by the dashed lines. The scale bar=1  mm.

**Fig. 8:**
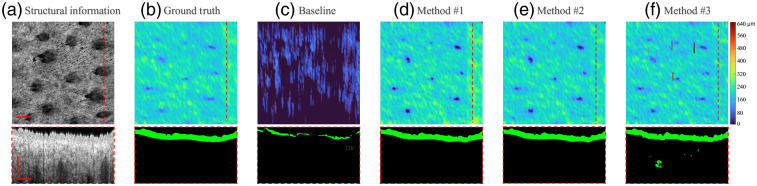
Segmentation results of OCT B-scan are in normal control site of patient #4. The representative B-scan OCT images and its corresponding *en-face* images are shown in (a), with expert annotations in (b) and baseline results in (c). The segmentation results for methods #1, #2, and #3 are shown in (d)–(f), respectively. The location of the selected cross-sectional images was indicated by the dashed lines. The scale bar=1  mm.

For illustration, four representative B-scan images from patient #4 throughout the healing process (session I, session II, session III, and control normal) were selected. *En-face* OCT structural images and selected B-frame OCT images [Figs. 5–8(a)] were also provided. Figures 5 and 8(d)–8(f) show the segmentation performance of the representative B-scan images using methods #1 to #3 over the healing timeline, alongside its corresponding epidermis thickness mapping. A color code was used to signify the thickness range of 0 to 640  μm (0 to 80 pixel). For qualitative analysis, the segmentation outputs of various approaches were visually examined and compared with the equivalent manual ground truth [Figs. 5–8(b)] and baseline [Figs. 5–8(c)].

Overall, upon qualitative assessment of the baseline results, which are directly applied to the pretrained rodent model to predict human data, there was worse agreement between the baseline and predicted structure for all the sessions of burning wound. Only a thin, discontinued layer in the outermost surface of skin can be predicted by the model, which can be clearly seen by segmentation result and its corresponding epidermis thickness mapping [Figs. 5–8(c)].

For segmentation performance in normal site using unsupervised learning (method #3), it was subjectively consistent with the manual segmentation, despite the occurrence of a few outliers and misclassified “islands” [see Figs. 8(b) and 8(f)]. Noticeable deviations from ground truth could be observed using method #3 in the region of the burning wound. The area with severe “under-prediction” of epidermis could be associated with lower contrast between epidermis and dermis in the burning lesion regions [see Figs. 5–7(f)]. The feature of the epidermis mapping in Figs. 5–6(f) is diffuse and vague compared with the baseline results due to the low prediction accuracy in the lower edge of the epidermis.

As for applying the semisupervised method #1 (one labeled image per volume) and #2 (three labeled images per volume), it presents a higher visual agreement with ground truth. Fewer hollow and outlier effects in the predicted epidermis region can be clearly seen in Figs. 5 and 8(d)–8(e). Performance of the scab segmentation using method #1 has an obviously low visual agreement with ground truth, which can be observed in Figs. 7(b) and 7(d). The epidermis segmentation using methods #1 and #2 seems to show a similar performance.

### 4.2 Quantitative Segmentation Results Analysis

Mean IOU, DSC, ASSD, and HD were used as metrics for the evaluation of the segmentation performance in validation dataset using baseline result and methods #1 to #3 (Table 1).

Intersection over union (IOU): IoU evaluates the fraction of correctly predicted instances of the validation datasets. The IoU is 0 for no overlap and 1 for perfect overlap. Given a number of true instances GT and the number of predicted instances Pred by a method, IoU can be defined as

$$\mathrm{IoU}(\mathrm{GT},S)=\frac{\left|\mathrm{GT}\cap \mathrm{Pred}\right|}{\left|\mathrm{GT}\cup \mathrm{Pred}\right|}.$$ (4)

Dice similarity coefficient (DSC) is a spatial overlap measure for segmentation, which is similar to IoU. DSC is 0 for no overlap and 1 for perfect overlap. It is related to IoU, which can be defined as

$$\mathrm{DSC}=\frac{2*\mathrm{IoU}}{1+\mathrm{IoU}}.$$ (5)

Average symmetric surface distance (ASSD): The size of the segmented areas has an effect on the DSC since misclassifications have a stronger impact on smaller areas than on larger ones. Therefore, we additionally use ASSD in this work. Let NS={p0,…,pn1} and NGT={q0,…,qn2) be subsets of a predicted segmentation S and a ground truth GT with NS⊆Pred and NGT⊆GT containing surface points. The surface distance SD between SP and SG is then defined as

$$\mathrm{SD}(S_{p},S_{G})=\overset{n2}{\underset{i=0}{\sum }}\;\min{\left\|p_{j}-q_{i}\right\|}_{2}.$$ (6)

The surface distance can then be used to determine the ASSD

$$\mathrm{ASSD}=\frac{\mathrm{SD}(S_{P},S_{G})}{2n_{2}}+\frac{\mathrm{SD}(S_{P},S_{G})}{2n_{1}}.$$ (7)

Hausdorff distance (HD) is also reported for outlier detection

$$\mathrm{HD}=\max[\mathrm{SD}(S_{P},S_{G}),\mathrm{SD}(S_{P},S_{G})].$$ (8)

From Table 2, the baseline result achieved the worst segmentation performance according to the four metrics, which differs significantly from those of other methods. The quantitative results are consistent with the cross-sectional segmentation result we showed before which had more topological error. All the methods show better segmentation performance in normal human skin than the skin disorder in terms of four metrics.

**Table 2:** Quantitative analysis of the comparative self-supervised strategy for sessions I, II, III, and control normal data in human. The value in bold indicates the best performance.

	Mean IOU	DSC	ASSD	HD (μm)
Quantitative result (burning wound session I)				
Baseline	0.332 ± 0.117	0.374 ± 0.127	50.30 ± 15.88	254.91 ± 105.40
Method #1	0.876 ± 0.032	0.883 ± 0.047	17.99 ± 10.49	120.94 ± 49.85
Method #2	**0.924 ± 0.024**	**0.914 ± 0.036**	**17.58 ± 5.29**	**115.30 ± 45.49**
Method #3	0.807 ± 0.053	0.812 ± 0.089	22.59 ± 8.50	183.83 ± 59.65
Quantitative result (burning wound session II)				
Baseline	0.340 ± 0.167	0.288 ± 0.134	60.59 ± 22.35	271.88 ± 160.50
Method #1	0.854 ± 0.024	0.863 ± 0.044	20.59 ± 8.59	84.55 ± 47.40
Method #2	**0.903 ± 0.011**	**0.929 ± 0.025**	**18.52 ± 4.50**	**67.63 ± 20.50**
Method #3	0.775 ± 0.032	0.777 ± 0.031	24.29 ± 10.30	145.82 ± 72.40
Quantitative result (burning wound session III)				
Baseline	0.433 ± 0.195	0.453 ± 0.211	75.40 ± 33.50	235.23 ± 165.92
Method #1	0.835 ± 0.068	0.859 ± 0.069	23.02 ± 5.05	89.40 ± 35.10
Method #2	**0.879 ± 0.092**	**0.882 ± 0.064**	**21.10 ± 4.99**	**74.92 ± 29.84**
Method #3	0.744 ± 0.073	0.737 ± 0.101	30.20 ± 10.51	157.12 ± 69.20
Quantitative result (normal site)				
Baseline	0.538 ± 0.082	0.604 ± 0.018	30.85 ± 10.72	186.54 ± 148.98
Method #1	0.930 ± 0.015	0.923 ± 0.031	16.50 ± 4.09	82.50 ± 16.28
Method #2	**0.942 ± 0.014**	**0.935 ± 0.025**	**15.59 ± 4.02**	**72.53 ± 18.30**
Method #3	0.925 ± 0.012	0.914 ± 0.042	16.83 ± 4.20	108.85 ± 40.51

IOU: intersection over union, DSC: Dice similarity coefficient, ASSD: average symmetric surface distance, HD: Hausdorff distance.

Significant improvements in all the metrics of the semisupervised learning (methods #1 and #2) compared with the unsupervised strategy (method #3) could be observed. It is noted that the improvement rate of HD score using methods #1 and #2 is >30% compared with method #3 in sessions I and II. This means our proposed semisupervised learning can significantly improve the robustness against the outliers in prediction of lesion site. The semisupervised learning using method #2 outperforms method #1 in all the skin conditions with both higher IOU and DSC scores, and lower ASSD and HD score. However, with a few exceptions, only a minor improvement happened according to IOU, Dice, and ASSD (increasing rate is <8%). With more labeled data for semisupervised learning, significant improvements (25.0%) are emphasized for HD score in the burning wound session II.

### 4.3 Epidermis Thickness Measurement

The deviations (mean ± standard deviation) between mean epidermis thickness acquired by methods #1 and #2 against ground truth for each patient are summarized in Table 3. The range of minimal and maximal error for method #1 is 5.4 to 21.9  μm, which represents ∼1 to 3 pixel error in OCT images. For individuals, maximum mean deviation is 14.0  μm, which is exhibited as clinically acceptable. Deviations between the mean epidermis thickness value using method #2 against ground truth were lower, which is in the range of 1.1 to 16.1  μm. Basically, only a slight improvement is shown with more labeled data for semisupervised learning.

**Table 3:** Epidermis thickness deviation evaluation for each patient using our proposed method compared with manual ground truth. C-control data (healthy site), S1-burning wound session I, S2-burning wound session II, and S3-burning wound session III.

Patient No.	#2				#3				#4			
Session	C	S1	S2	S3	C	S1	S2	S3	C	S1	S2	S3
Ground truth (GT) (μm)	244.9	157.0	174.6	209.2	210.6	194.5	161.9		180.3	475.3	387.4	342.5
Method #1 (μm)	239.5	147.3	188.6	196.6	222.5	208.5	155.9		185.7	492.0	365.5	325.6
Method #2 (μm)	239.2	150.5	184.3	202.6	201.3	204.8	157.4		183.5	487.6	371.6	331.1
Mean deviation between method #1 and GT (μm)	10.4 ± 3.5				10.6 ± 3.6				14.0 ± 6.5			
Mean deviation between method #2 and GT (μm)	7.1 ± 1.6				8.0 ± 2.8				9.7 ± 5.1			
**Patient No.**	**#8**				**#13**							
Session	C	S1	S2	S3	C	S1	S2	S3				
GT (μm)	150.0	198.5	258.7	342.5	241.5	282.3	305.4	277.8				
Method #1 (μm)	142.5	214.2	274.2	325.6	233.1	271.6	326.3	265.1				
Method #2 (μm)	151.1	210.2	269.2	331.1	237.2	275.0	321.5	266.8				
Mean deviation between method #1 and GT (μm)	13.9 ± 3.4				13.2 ± 5.0							
Mean deviation between method #2 and GT (μm)	8.8 ± 4.7				9.7 ± 4.7							

## 5 Discussion

Recently, there is increasing research activity that utilizes rodent models to mimic human skin response,^41^^–^^44^ especially in wound healing,^45^ skin inflammation,^46^ and nonmelanoma skin cancer.^47^ Insufficient clinical human skin OCT datasets highlight the necessity for a technique that can leverage the rodent annotation model to better serve the human datasets. Our study demonstrates the feasibility of the semisupervised pixel-level segmentation of the epidermis and scab region in human data using the mouse pretrained model. To our knowledge, this is the first study that addresses the automatic segmentation in human skin by making full use of the knowledge, learned from datasets acquired from rodent models.

We presented a method based on the semisupervised learning framework with a multitask U-Net model. This method allows one to build representations that capture both contour and intensity information in the source domain and leverage the information to the target domain by finetuning the model, which only required limited quantities of labeled data. Having successfully tested and validated on target domain (19 acute burning wound data volumes from five patients over healing timeline), our proposed method can localize the epidermis and scab region accurately compared with the manual segmentation by experts. Besides, according to the evaluation metrics, it outperforms the baseline model and unsupervised model. In addition, the proposed robust segmentation framework can be extended to the 3D segmentation of OCT volumes, and the epidermal thickness mapping can be provided from the region of interest.

Our proposed semisupervised deep learning method was able to correct some of the false positives and improve the robustness against the outliers. It is attributed to the multitask model harnessing additional context information available around the contour of the structure, assisting the delineation of the target region smoother and more intact. Moreover, the result suggests that the contour information between the layers (epidermis-dermis and scab-epidermis) yields an essential information to bridge the difference between rodent and human datasets. The proposed model tends to emphasize the contour information to detect the local signal drop between epidermis and dermis, which is always presented in a variety of skin abnormalities.^48^ Furthermore, it could be shown that the result has high variability using unsupervised learning. Many false-positive predicted regions and outliers are more pronounced in the skin-damaged regions with lower contrast. Due to the relatively low similarity of intensity features in the same anatomy structure at different species, the unsupervised learning will cause severe drift problems.

The proposed semisupervised learning shows numerous advantages: (1) With the help of a multitask pretrained model, the segmentation performance of the self-supervised learning can be visibly improved on small-scale labeled clinical datasets. (2) According to the quantitative result, the semisupervised model achieved the best results on the datasets from different sessions of the six patients, suggesting a good capability of robustness and generalization. (3) The proposed model significantly improves the work efficiency in clinics, which makes full use of the rodent data (source domain), and only one random labeled image per volume is demanded to achieve a satisfying result. (4) It offers a computationally inexpensive and faster segmentation framework that only takes 70 ms to segment one OCT image. Additionally, segmentation speed for each volume (800 B-frames) is ∼5.6  s, which is fast enough for most clinical applications.

Several limitations still exist in our study. We were unable to offer additional validation to our model by comparing it to the histology datasets since providing a 3D histological information is a difficult endeavor. Moreover, it is worth noting that the human datasets used in the current study were from patients with acute burned wounds, and therefore further work is required to examine the performance of the proposed method in cases of other skin disorders, like skin inflammation, basal cell carcinoma, chronic wound, etc.

In conclusion, we presented a semisupervised representation learning, a model that can transfer the structure annotation in the mouse domain based on U-Net encoding, to guide the segmentation of clinical data in humans. Extensive comparison of the burning wound dataset between different self-supervised strategies demonstrated the outstanding segmentation performance of our method. We believe the proposed method could serve to promote the usage efficiency of rodent models and promises to aid in the clinical assessment and treatment planning of skin diseases.
